# Study on thermal insulation cement and its thermal insulation characteristics for geothermal wells

**DOI:** 10.1038/s41598-023-30614-y

**Published:** 2023-03-13

**Authors:** Wei Zhou, Chengwen Wang, Renzhou Meng, Zehua Chen, Haoxin Lu, Jialun Chi

**Affiliations:** 1 Key Laboratory of Unconventional Oil and Gas Development, China University of Petroleum (East China), Qingdao, 266580 China; 2 College of Petroleum Engineering, China University of Petroleum (East China), Qingdao, 266580 China; 3 CNPC Engineering Technology R&D Company Limited, Beijing, 102206 China

**Keywords:** Composites, Mechanical properties

## Abstract

Reducing the heat loss in wellbore is the key for efficient development of geothermal resource. It is a reliable solution to establish a long-term stable wellbore with good thermal insulation through cementing. In this paper, the cement-based composite thermal insulation material was prepared by using cement as the cementing material, hollow glass beads, foaming agent and stabilizer as main raw materials, and other conventional admixtures. Foams and hollow glass beads can introduce gas with low thermal conductivity into cement, so as to improve the thermal insulation of composite material. Foams are produced by chemical forming process, using foaming agent, which is prepared according electrochemistry and thermodynamics, and the foam stabilizer helps foam distribute in cement slurry stably and uniformly. 10–13% hollow glass beads can significantly reduce the thermal conductivity of hardened cement, without significant adverse effects on the rheology and strength of the material. The thermal conductivity of the composite thermal insulation material can be as low as 0.2998 W·(m·K)^−1^, which is 62% lower than that of conventional cement, while the compressive strength is 6.10 MPa, meeting the engineering requirement. A thermal-conductivity prediction method is proposed correspondingly based on Maxwell model, and the prediction error of the newly established model is within 2%. This research can provide technical support for efficient development of geothermal resources.

## Introduction

With the continuous growth of global energy demand and the continuous reduction of conventional energy reserves, various alternative energy sources have gradually attracted extensive attention. Geothermal energy is widely distributed in the world and is rich in reserves. It is a green and renewable resource. In recent years, it has been developed in many countries^1^. As a tool for the development of geothermal resources, the geothermal wells should have high quality for the effective utilization of geothermal energy^2^. However, there is a large amount of heat loss in the flowing process of high-temperature fluid from wellbore bottom to surface, which is a big bottleneck restricting the efficient utilization of geothermal wells^3,4^. It is a feasible solution to establish a long-term stable wellbore with high thermal insulation effect, which exerts higher requirements for the thermal insulation and mechanical properties of cementing fluid^3,4^.

Cementing fluids are mostly cement-based materials. In recent years, cement-based thermal insulation materials have been widely studied and applied in the field of construction, but they are rarely used in the field of oil and gas wells, especially geothermal wells^5^. On the one hand, insufficient attention has been paid to the wellbore insulation in the process of geothermal well exploitation; on the other hand, there is a lack of relevant research on the insulation cement slurry suitable for geothermal wells^6^. Foamed cement is prepared by introducing bubbles into cement-based materials by means of chemical foaming and/or physical foaming. It has low density and good thermal insulation performance, and has a potential for geothermal well insulation^7^. However, the preparation of nitrogen using mechanical method requires a series of equipment and associated systems^5,8^, and the corresponding work process is complex and the cost is high. Nitrogen prepared using chemical method has the advantages of simple operation and low cost, but generally the gas production efficiency of chemical foaming agent is low^9,10^. Hollow glass beads have many hollow structures, low thermal conductivity and its preparation process is simple^11–13^. However, in order to achieve an ideal thermal insulation performance, the use of hollow glass beads requires more dosage. This will impose a more significant impact on the performance of cement slurry, and the cost of glass beads is high^14^.

The research on the thermal conductivity of composites provides a good guidance in the preparation of thermal insulation cement system^15,16^. Maxwell model can be used to calculate the thermal conductivity of cement composites, with the premise that the dispersed phase particles are evenly distributed in the continuous phase medium, and that no interaction occurs with each other^17^. Also, an assumption that the particle shape is spherical and randomly distributed is necessary. In porous hollow materials, pores are mostly cavities with different shapes^18^. The thermal conductivity of this structure will depend on the thermal conductivity of solid particles and pores, as well as the size, shape and distribution of pores. When studying the thermal conductivity model for concrete, Zhang Weiping et al.^19^ divided the thermal conductivity prediction model of two-phase composites into three categories: series parallel model without considering interface thermal resistance; Maxwell model without considering interfacial thermal resistance and its extension; Maxwell model considering interfacial thermal resistance and its extension. However, the thermal conductivity model of multi-component composite system containing foamed cement slurry still lacks specific research.

Based on the principle of nitrogen production using chemical method, the chemical nitrogen foam additive is selected. The effect of nitrogen foam and hollow glass beads on the strength and thermal conductivity of hardened cement is analyzed. The thermal conductivity of cement slurry is analyzed, and the prediction model of thermal conductivity of cement-based composites is established. Based on this, a novel insulation cement slurry has been developed and successfully applied to ultra-deep heavy-oil well for the first time. This study provides a reference for the design and engineering application of cementing fluid in geothermal wells.

## Methods

### Experimental materials

Class G oil well cement (Jiahua, Sichuan), polymer retarder HX-36L (AMPS/AA/DMAA/IA copolymer, house made), dispersant QS-20S (Chengdu Omax petroleum technology co., LTD.), Nano stabilizer NS-2 (Nano-silicate particles, house made). Foaming agents for producing nitrogen using chemical method are self-made in the laboratory.

As known, the oxidation value of nitrogen can be positive (+ 5, + 4, + 3, + 2, + 1), zero or negative (-1, -2, -3). The reaction of nitrogen generation is redox reaction, thus the oxidation number of nitrogen will be reduced to zero. Based on that, the reactants containing different valence nitrogen were tested, and the reactants combination with the lowest Gibbs free energy was selected as the foaming agent according to the basic principle of chemical reaction. Further, the reaction capacity and the overall volume of nitrogen produced were tested through experiments, considering the effects of reactants and products on the properties of cement slurry. As such, the efficient chemical gas-generating agent, i.e., NGR system: NGR-I and NGR- II were developed. NGR-I is a kind of white fine powder while NGR-II is a kind of colorless liquid, and they are soluble in water. Once the two materials meet, they can react to release nitrogen.

Foamed cement slurry is a thermodynamically unstable system. Foam stabilizer is needed to stabilize the foaming system^9,20^. The free fluid of cement slurry has a high salinity and a high alkalinity, thus requiring high salt and alkali resistance of foam stabilizer^10^. Therefore, an efficient foam stabilizer SC-1 with high alkalinity and salinity resistance was developed, based on the temperature and alkali resistance of protein foam stabilizer and the film-forming characteristics of surfactant.

**Figure 1:**
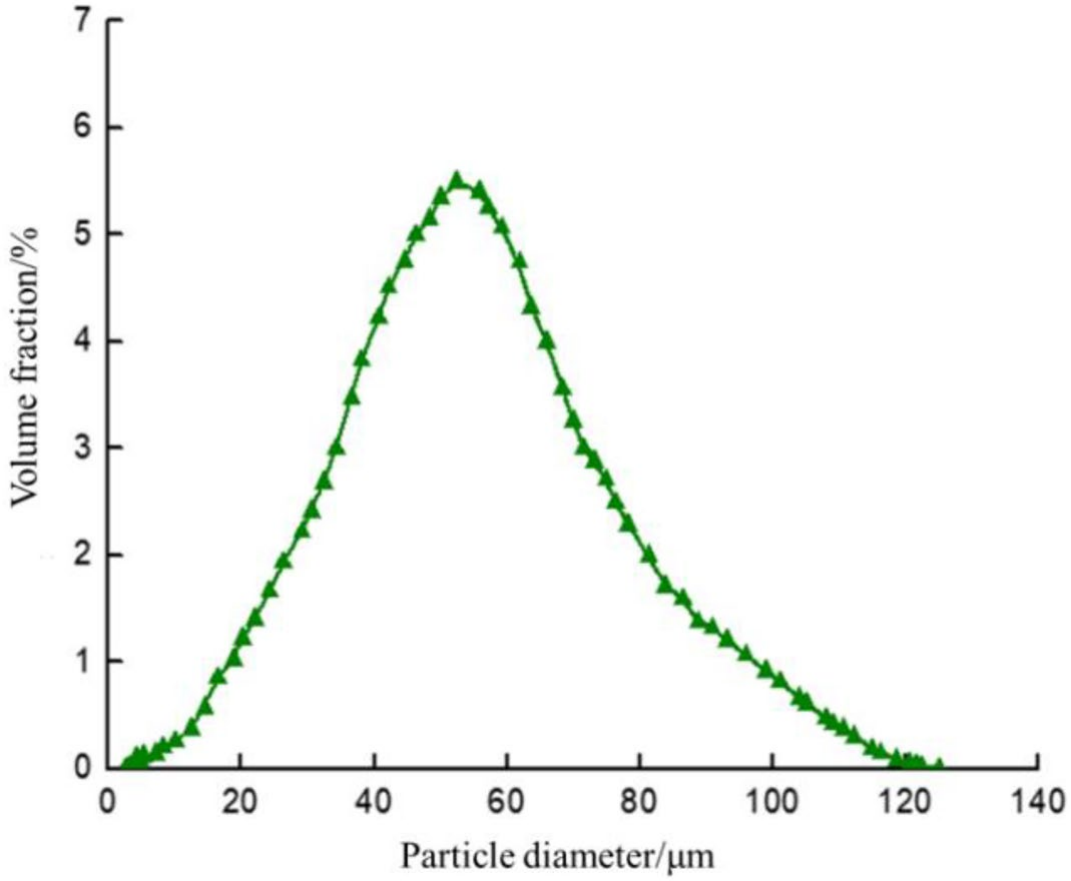
Particle size distribution of hollow glass beads.

Hollow glass beads are hollow spheres made from alkali lime borosilicate glass. They show characteristics of low density, small particle size and high-pressure bearing capacity. In recent years, they are widely used in density reduction and heat preservation^11–13^. The main performance parameters of hollow glass beads used in this study are: the real density is 0.35 g/cm^3^, the compressive strength is 69 MPa; The particle size is mainly distributed in the range of 15–85 μm, while the maximum particle size (diameter) is 122.73 μm and the average particle size is 52.55 μm. It has a uniform particle-size distribution, which is shown in Fig. 1.

The water used in this paper is tap water. Sodium hydroxide (NaOH), Sodium Dodecyl Benzene Sulfonate (SDBS) and other drugs were purchased from Sinopharm Group and were chemically pure.

### Experimental method

#### Cement slurry preparation

1. Conventional cement slurry: the conventional cement slurries were prepared according to API RP 10B-4-2013^21^.
2. According to the field characteristics of chemical-induced nitrogen foam cement slurry, the chemical-induced nitrogen foamed cement slurry was prepared by injecting, mixing and stirring in a closed system. Firstly, the self-made closed stirring device was opened and filled with a certain quantity of water and foam stabilizer; Then, at a string rate of 4000 rpm, the oil-well cement and foaming agent NGR-I which have been mixed evenly were added within 15 s; Keep the closed mixing device in a closed state and inject a suitable volume of foaming agent NGR- II in 5 s, continue stirring 35 s at 12,000 rpm, and finally the whole closed mixing container should be completely filled with foamed cement slurry.

#### Nitrogen production evaluation

The gas production of the foaming agents was evaluated by water displacement method considering the very low solubility of nitrogen in water.

#### Performance tests of cement slurry

1. Density test. The density of cement slurries was tested according to API 10B-4–2004 “Recommended Practice on Preparation and Testing of Foamed Cement Slurries at Atmospheric Pressure” by API densimeter.
2. Rheological property. The rheological property of cement slurries was tested according to API 10B-4–2004 by API six-speed viscometer. The rheological properties of cement slurries are characterized by the fluidity index *n* and the consistency coefficient *K*. The calculation method is as follows:
  1 $$n = 2.092\lg \left( {\frac{{\theta_{300} }}{{\theta_{100} }}} \right)\quad K = \frac{{0.511\theta_{300} }}{{511^{n} }}$$
  where: *θ*_300_ and *θ*_100_ are the reading of the viscometer at 300 and 100 rotor speed, respectively, when cement slurries are tested; *n* represents the flow index, and a higher value of *n* indicates a better flow performance of cement slurry; *K* is the consistency coefficient, and a higher value of *K* indicates that the cement slurry is thicker.
3. Water loss. According to API 10B-4-2004, the cement slurry should be cured for 20 min using atmospheric pressure or pressurized thickener, and then the water loss of cement slurry was tested by high-temperature and high-pressure loss meter.

#### Performance tests of hardened cement

1. Compressive strength and elastic modulus. Uniaxial compression test system was used to evaluate the compressive strength of hardened cement. Testing samples were prepared using cube molds with specifications of 50.8 mm × 50.8 mm × 50.8 mm. The sample was placed on the support block of the uniaxial compression tester. Before testing, ensured that the spherical base with the support block can be tilted freely, and there was no cushion or firm cushion between the sample and support block. The stress—strain curve of hardened cement was recorded by applying load gradually. The compressive strength of cement blocks is the ratio of the maximum stress to the cross-section of cement blocks.
2. Thermal conductivity coefficient. The thermal conductivity of cement block was measured by a transient hot-wire thermal-conductivity tester (Hot-wire method, TC3000E, Xi'an Xia Xi Electronic Technology Co., Ltd.). The specific operation steps of the instrument were as follows: The sensor was sandwiched between two samples, and the supporting weight was placed on the top of the sample; The Heat Balance of the sensor was detected before test (the temperature can be considered to be in balance when the temperature detection volatility is ≤ 0.05/10 min); In the software, appropriate test conditions or substance name was selected, and then the thermal conductivity was measured.
3. Coefficient of thermal expansion. For cement-based materials, the coefficient of thermal expansion (CTE) is the main parameter characterizing their thermal expansion properties. The CTE of cement is related to the moisture content, internal relative humidity, porosity, and development of the cement hydration products. Recently, most of the studies have been focused on the thermal expansion property of the hardened cement paste under the water-saturated condition. The CTE was tests according to the method of Yuhuan Bu^22^.
4. Scanning Electron Microscope. The cement samples were pasted to a copper sample holder with conductive adhesive, gilt in a vacuum, and then observed under Zeiss EVO 25 SEM (Zeiss, Germany)

## Results and discussion

### Chemical-induced nitrogen-foam additive

#### Foaming agent for producing nitrogen using chemical method

**Figure 2:**
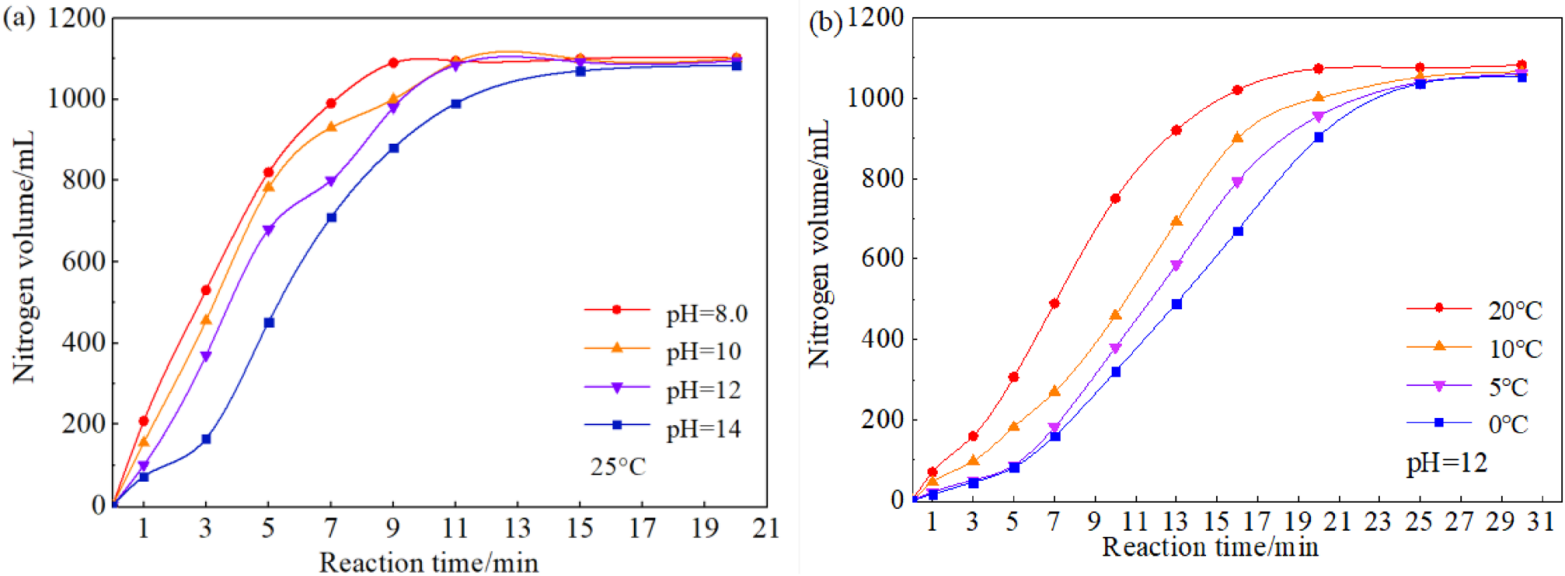
Effect of different conditions on nitrogen gas volume: (**a**) pH; (**b**) temperature.

The free water in cement slurry is usually a saturated Ca(OH)_2_ solution, which provides a highly alkaline environment and may affect the redox reaction of nitrogen production^9^. Therefore, the variation of nitrogen volume production (by NGR system) with time under different pH value is tested. The experimental formula is: 6.0 g NGR-I + 2.2 g NGR-II + 100 g NaOH solution (pH is controlled by adjusting NaOH concentration), and the generated nitrogen is collected using the water displacement method, as shown in Fig. 2a.

The results show that the foaming agent NGR system can still produce nitrogen even under the condition of high alkalinity, adapting to the alkaline environment of cement slurry and realizing the purpose of nitrogen chemical-induction; The pH value has a great influence on the initial reaction rate of nitrogen production. The smaller the pH value, the faster the initial rate of nitrogen production by the NGR system. The final volumes of nitrogen produced are 1102, 1100, 1095, 1083 mL, respectively, when the pH values are 8, 10, 12 and 14, indicating that the final volume decreases slightly with the increase of pH.

Figure 2b shows the effect of temperature on nitrogen production by foaming agent. As can be seen, the foaming agent NGR system can react at a low temperature (0 °C) to produce nitrogen quickly, thus it can be used in a low-temperature environment. The temperature has a significant effect on the initial reaction rate of nitrogen production. The amount of nitrogen produced at the beginning of the reaction is small. However, due to release of heat, the reaction will accelerate with the progress of the reaction. The reaction rate will accelerate significantly and the volume of nitrogen will increase greatly within 5 min. The final volumes of nitrogen produced are 1082, 1065, 1060, 1052 mL, respectively, when the temperatures are 20 °C, 10 °C, 12 °C and 4 °C, indicating that the final volume also decreases slightly with the decrease of temperature.

#### Foam stabilizer

The half-life of foaming liquid was used to evaluate the effect of foam stabilizer. As the foam is a thermodynamically unstable system, it would continue to be broken and the liquid film would continue to collapse as the standing time becomes longer. The half-life of the foam liquid is defined as the time when 50% foam is broken^23^. The foam-stabilizing effect of SC-1 was tested at 25 °C. The results show that, the initial volume of the foam was 580 mL, and the half-life of the liquid was 120 min. The volume of the foam was still as high as 565 mL at the half-life of the liquid, showing excellent foam stabilization ability.

**Figure 3:**
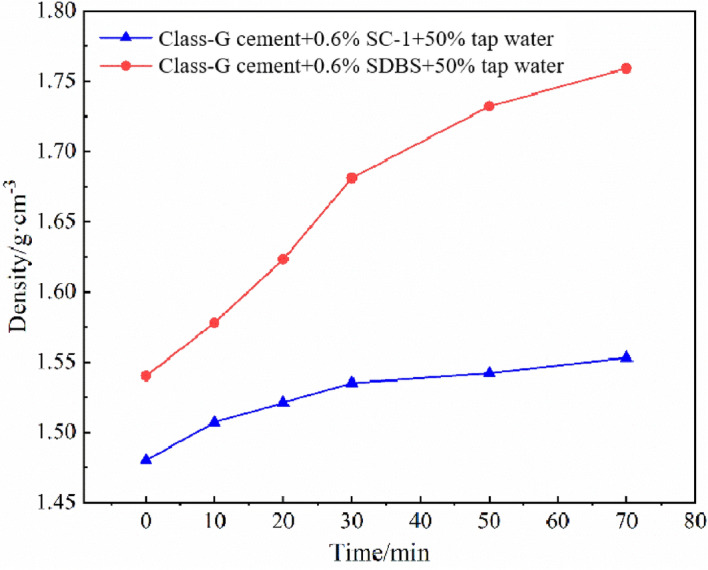
Variation of density of foamed cement slurry with time.

The density of foamed cement slurry stabilized by different stabilizer was tested at 25 °C, and its foam-stabilizing capacity was further analyzed. The cement slurry system used is written below: Class-G cement + 0.6% SC-1 foam stabilizer / sodium dodecylbenzene sulfonate (SDBS) + 50% tap water. The results are shown in Fig. 3: the density of cement slurry with addition of foam stabilizer SC-1 increases quickly within 10 min, and then the rate of density increase slows down, and the increase trend is smaller after 30 min. The cement slurry density increases from 1.48 to 1.553 g/cm^3^ after standing for 70 min, with an increase rate of 4.9%, and the system is relatively stable; In contrast, the foamed cement slurry with SDBS as foam stabilizer had a larger foam diameter and poor foaming stability, and the density of cement slurry increases from 1.54 to 1.759 g/cm^3^ after being placed for 70 min, and the density increase rate was 14.22%. The test results fully show that foam stabilizer SC-1 has very good foam-stabilizing ability.

#### Effect on the properties of cement

The effect of nitrogen foaming additive on the strength and thermal conductivity of cement paste was analyzed, as shown in Table 1.

**Table 1:** Effect of foam additive on strength and thermal conductivity of hardened cement.

NGR/%	SC-1/%	Density before and after foaming/g·cm^−3^	Compressive strength /MPa		Elasticity modulus /GPa		CTE/10^–6^ °C^–1^		Thermal conductivity/W·(m·K)^−1^
			60 °C	100 °C	60 °C	100 °C	60 °C	100 °C	
0	0	1.8	26.74	28.93	9.8	10.2	11.7	9.5	0.7577
0.7	2	1.8/1.1	17.71	18.34	8.6	8.9	8.4	7.3	0.4149
1.0	2	1.8/0.9	16.92	17.47	7.7	8.0	6.9	5.6	0.4103
1.5	2	1.8/0.8	13.68	14.15	5.7	6.1	5.4	4.1	0.3998
2.0	2	1.8/0.7	8.0	8.9	4.1	4.5	3.1	2.2	0.3933

The percentage of foaming agent and foam stabilizer is the mass percentage of Class G cement ash.

It can be seen that, the volume of nitrogen production in the cement slurry continues to increase with the dosage of NGR foaming agent increasing, and the slurry density gradually decreases correspondingly after foaming. When the dosage is 2%, the surface density can be reduced to 0.7 g/cm^3^. The compressive strength test shows that the 48 h compressive strength of harden cement also decreases with the increase of NGR dosage. This is because the foam affects the integrity of the cement structure, resulting in the reduction of the mechanical strength of the cement. The expansion coefficients of cement at 100 °C is less than that at 60 °C, which can be attributed to the shrinkage of cement volume caused by the dehydration of C–S–H gel and Ca(OH)_2_ in the slurry at higher temperature. Moreover, the coefficient of thermal expansion is slightly reduced after adding the Foaming agent within 100 °C. On the whole, the addition of admixture has little influence on the expansion coefficient of hardened cement within the range of test temperature, and will not have significant influence on the performance of hardened cement and the structure of cement ring in the later stage.

And, the analysis of mechanical properties and expansion coefficient of cement, as shown in Table 1, shows that the compressive strength of harden cement is positively correlated with the CTE. Generally, CTE of hardened cement with compact structure will be larger. Therefore, for the foamed cement, the CTE of the hardened cement is much lower than that of conventional cement.

The thermal conductivity test shows that the foamed cement has good thermal insulation performance. When the NGR dosage is 2%, the thermal conductivity of hardened cement is reduced to 0.3998 W·(m·K)^−1^, which is nearly 50% lower than that of conventional cement paste (0.7877 W·(m·K)^−1^). This indicates that the thermal conductivity of hardened cement can be significantly reduced by introducing foams.

### Influence of hollow glass beads on properties of hardened cement

#### Shear resistance of hollow glass beads

**Figure 4:**
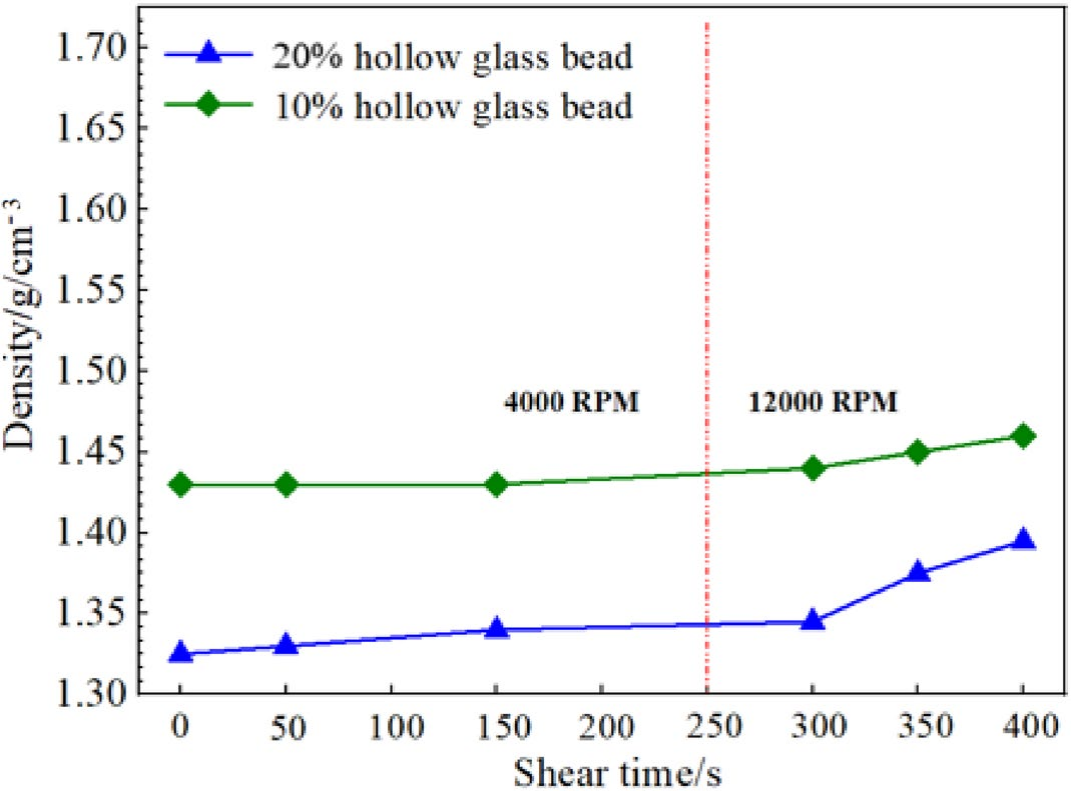
Variation of density of foamed cement slurry with shear speed.

Cement slurry is subjected to dynamic high-speed shear during preparation. In order to evaluate the shear resistance of hollow glass beads, a variable-speed stirrer was used to test the density variation law of low-density cement slurry with different dosage of hollow glass beads after continuous shear. The slurries were stirred for 50 s and 150 s at 4000 RPM, and first stirred for 250 s at 4000 RPM and then for a period of time at 12,000 RPM, respectively. Then the densities of those slurries were tested. As shown in Fig. 4, the hollow beads exhibit good shear failure resistance. They show good shear resistance at a low speed shear of 4000 RPM and a high-speed shear of 12,000 RPM when the dosage is 10%. However, the shear resistance of glass beads decreases slightly at high speed shear when the dosage is 20%, which may be because the probability of beads colliding with the blade increases greatly at high dosage, which increases the probability of breakage.

#### Effect on cement slurry and hardened cement performance

The effect of dosage of hollow glass beads on the performance of cement slurry, the mechanical properties and thermal conductivity was tested, and the results are shown in Table 2. The results show that hollow glass beads can effectively reduce the density of cement slurry. The net density of cement slurry is 1.71 g/cm^3^. With 5% beads addition, the density of cement slurry decreases rapidly to 1.53 g/cm^3^. When the dosage is 20%, the cement-slurry density can be reduced to 1.29 g/cm^3^. The rheological test shows that the rheological properties of cement slurry become worse with the addition of hollow glass beads gradually increasing from 0 to 15%. However, when its dosage exceeds 15%, hollow glass beads will show "spherical ball effect" due to their spherical characteristics, thus the rheological properties of cement slurry become better.

**Table 2:** Effect of hollow glass beads on cement slurry and hardened cement performance.

Bead dosage/%	Density/g·cm^−3^	Rheological parameters		Compressive strength (60 °C/48 h) /MPa	Elasticity modulus /GPa	CTE /10^–6^ °C^−1^	Thermal conductivity/W·(m·K)^−1^
		n	k				
0	1.71	0.9915	0.1444	25.49	9.5	10.7	0.7410
5%	1.53	0.7677	0.6047	24.34	9.1	9.4	0.6704
10%	1.44	0.8062	0.4556	22.36	8.4	8.2	0.6084
15%	1.32	0.9673	0.1422	20.64	7.5	6.7	0.5591
20%	1.29	0.9854	0.1231	18.74	6.8	5.1	0.4790

The percentage of bead is the mass percentage of cement ash.

The compressive strength test shows that the compressive strength of cement paste decreases gradually with the increase of the content of hollow glass beads. However, the decline was smaller than that of conventional cement slurry. The thermal conductivity test shows that hollow glass beads can significantly reduce the thermal conductivity of cement paste. In addition, the thermal conductivity of cement slurry gradually decreases with the increase of hollow glass beads; however, the thermal conductivity decreases less than that of foamed cement slurry.

### Combination of nitrogen-foam additive and glass beads

#### Cement performances

**Figure 5:**
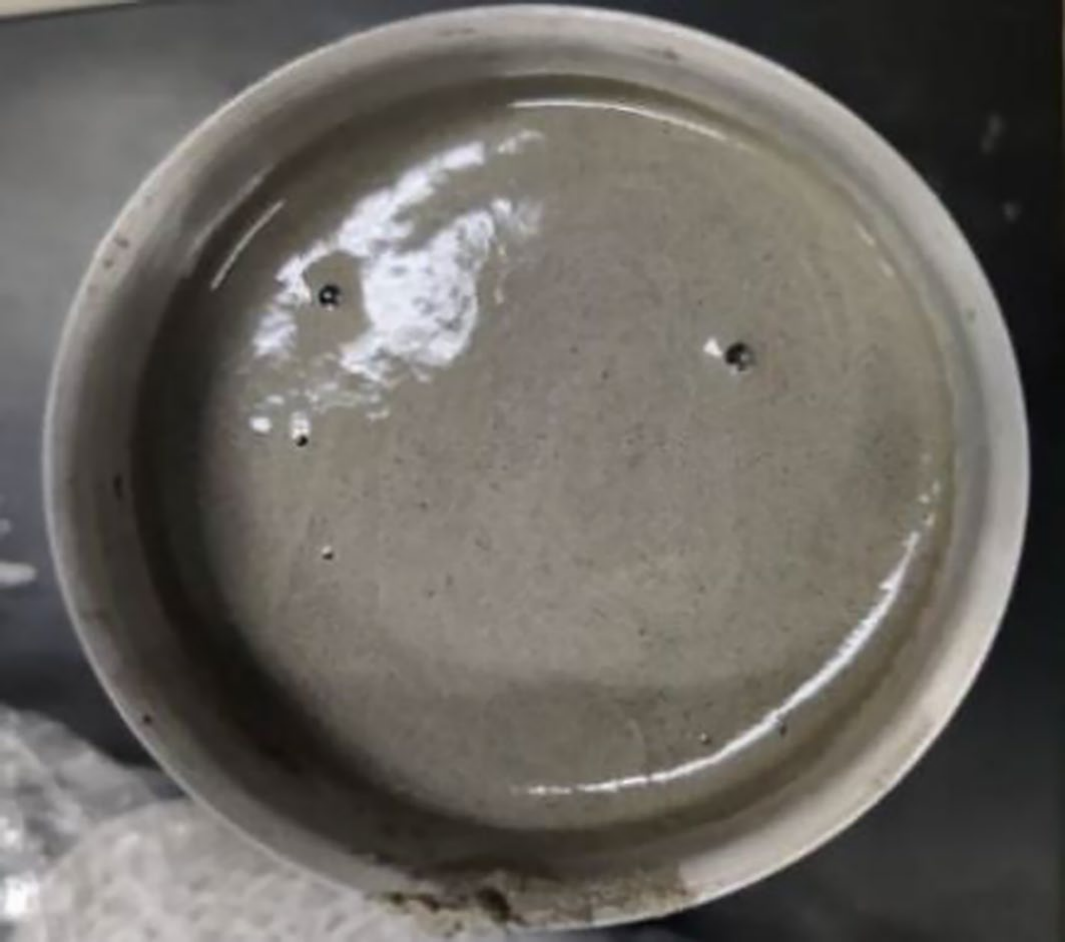
Appearance of cement slurry with the combination of chemical-induced nitrogen-foam additive and glass beads.

The chemical nitrogen-filled foamed cement has lower cost and causes lower thermal conductivity of harden cement, but the corresponding strength is low. Hollow glass beads can effectively reduce the thermal conductivity of cement paste, and have a relatively small impact on the compressive strength, but its price is high. As such, the effect of the combination of foam and hollow glass beads on cement performance was further studied. In order to ensure the good fluidity of cement slurry, proper amount of dispersant was added. The amount of foaming agent and foam stabilizer was 2%, and the water cement ratio was fixed at 0.44. The test results of cement paste density, rheological property, compressive strength and thermal conductivity are shown in Table 3, and the appearance of cement paste is shown in Fig. 5.

**Table 3:** Effect on the properties of cement.

Glass bead/%	Dispersant/%	Density before and after foaming /g·cm^−3^	Rheological parameters		Compressive strength (60 °C/48 h) /MPa	Elasticity modulus /GPa	CTE/10^–6^ °C^−1^	Thermal conductivity/W·(m·K)^−1^
			n	k				
5	0.2	1.58/1.1	0.9915	0.1444	7.6	3.7	2.8	0.4067
7.5	0.2	1.49/1.1	0.7677	0.6047	7.67	3.7	2.1	0.3973
10	0.2	1.43/0.9	0.8062	0.4556	6.82	3.1	1.4	0.3561
12.5	0.3	1.42/0.9	0.9673	0.1422	6.10	2.4	0.8	0.2998

It can be seen that, thermal conductivity of the harden cement decreases rapidly with the addition of glass beads, but the compressive strength also decreases to a certain extent. When the dosage of glass bead is 12.5, the thermal conductivity is as low as 0.2998 W·(m·K)^−1^, which is 62% lower than that of conventional cement (0.7877 W·(m·K)^−1^), while the compressive strength is 6.10 MPa, which still meets the engineering requirement. This indicates that the performance indexes of low density and high strength of cement can be achieved by the combination of chemical-induced nitrogen-foam additive and glass beads.

#### Microstructure

**Figure 6:**
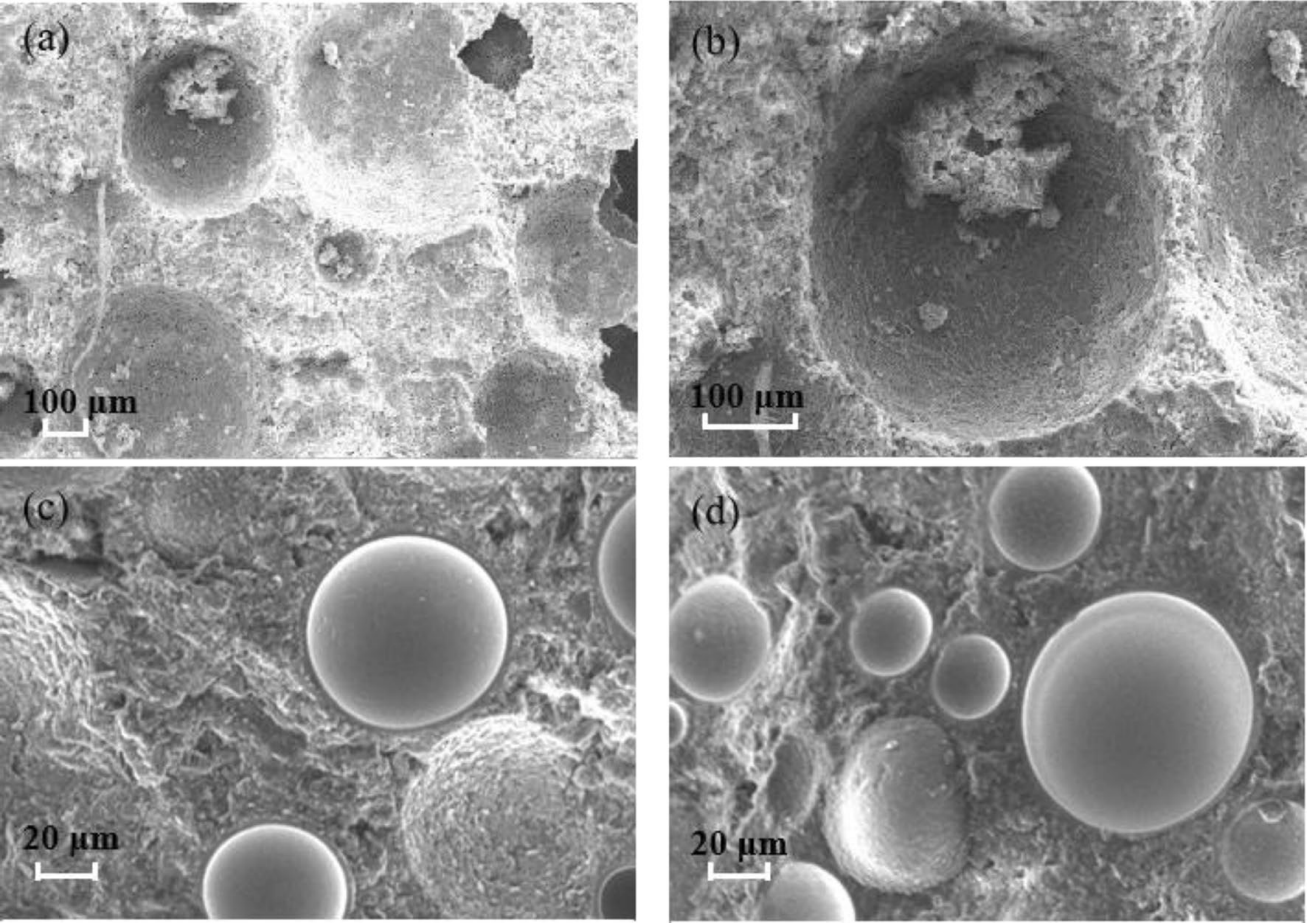
Microstructure of foamed cement: (**a**,**b**) is the hardened cement without hollow glass beads; (**c**,**d**) is the hardened cement with 12.5% hollow glass bead.

Different cement samples were selected to observe the microstructure of hardened cement. Figure 6 is the SEM of the section of hardened cement without hollow glass beads and with 12.5% hollow glass beads. It can be seen that in the hardened foam cement without hollow glass beads, the bubbles are uniform in size and exist in individual forms, even if the density is as low as 1.035 g/cm^3^. This not only improves the stability of the foamed cement slurry, but also makes the foamed cement with better properties such as small permeability, high compressive strength and good thermal insulation performance.

For the harden foamed cement with 12.5% hollow glass beads, the glass beads are evenly distributed in the hardened cement with little breakage. This indicates that the optimized hollow glass beads have good pressure resistance and the prepared slurry is stable, which can ensure that the hollow glass beads are completely and evenly dispersed in the hardened cement. This is because the main chemical composition of beads is silicate, which can react quickly with calcium released by cement hydration reaction to form calcium silicate hydrates. At the same time, it can be seen that the addition of hollow glass beads does not affect the stability and dispersion of bubbles in the hardened cement, and the pores exist independently and their distribution size is relatively uniform. The results show that the glass beads have good compatibility with the foam stabilizer. It can also be seen that some of the high-performance hollow glass beads are spalling, indicating that there is still weak bonding point between glass beads and cement, which may be one of the main reasons for the reduction of the compressive strength of cement after the addition of hollow glass beads.

#### Cement performances under different temperature/pressure

There is a large amount of nitrogen in foam cement. Due to the compressibility of nitrogen, the volume of nitrogen in foam cement will change significantly as the pressure/temperature changes. During cementing, there is usually an increase in pressure/temperature, and foamed cement paste will generally show an increase in density. In addition, the thermal conductivity of cement also needs to consider the volume proportion of thermal insulation material in slurry, so it is necessary to reflect the variation of nitrogen volume fraction in slurry in the annulus. In order to reflect the relationship between the density and the pressure of the foamed cement slurry system in this study, the density of the foamed cement slurry system under a certain pressure was tested, and the influence of the pressure change on the microstructure of foamed cement was discussed, so as to provide experimental basis for the reasonable design of the density and thermal conductivity of foamed cement slurry.

Since the temperature and pressure increase simultaneously with the increase of well depth, different combinations of temperature and pressure are determined as curing conditions for the cement slurry, and the density, thermal conductivity and compressive strength of the slurry are calculated and tested. The results are shown in Table 4. The results show that the thermal conductivity of cement increases with the increase of temperature and pressure. Generally speaking, the thermal conductivity of cement increases with the decrease of pore volume. According to the gas state equation, the gas volume is directly proportional to the temperature and inversely proportional to the pressure, and the pressure increases with wellbore depth much faster than the temperature. Therefore, the pore volume of foamed cement decreases with increasing well depth. However, its performance is still better than that of non-foaming cement slurry, indicating that foam cement still has good thermal insulation effect under high pressure.

**Table 4:** Cement performances under different temperature/pressure.

	20 °C/1 Bar	30 °C/8 MPa	40 °C/10 MPa	50 °C/15 MPa	60 °C/20 MPa
Nitrogen density/g·cm^−3^	0.0012	0.0049	0.0067	0.0089	0.01
Slurry density/g·cm^−3^	0.9	1.43	1.48	1.51	1.53
Nitrogen volume fraction/%	43	11	8	6	5
Thermal conductivity/W·(m·K)^−1^	0.2599	0.3413	0.3564	0.3667	0.3719
Compressive strength /MPa	6.2	6.8	7.4	7.8	8.2

### Thermal conductivity model of composite cement slurry

#### Evaluation of thermal conductivity model for hollow glass bead cement slurry

As for the heat conduction of filled composites, Maxwell model and Russell model are selected to simulate the heat conduction process in hardened cement. Maxwell model and Russell model can be expressed by Eqs. (2) and (3), which are written below respectively:

2 $$\lambda c=\lambda m\left[\frac{2Vf\left(\frac{\lambda f}{\lambda m}-1\right)+(\frac{\lambda f}{\lambda m}+2)}{Vf\left(1-\frac{\lambda f}{\lambda m}\right)+(\frac{\lambda f}{\lambda m}+2)}\right]$$

where λ_c_ is the thermal conductivity of the composite material, λ_f_ is the thermal conductivity of the uniformly distributed filling phase, λ_m_ is the thermal conductivity of the filling matrix, and V_f_ is the volume fraction of the spherical filling phase.

3 $$\lambda ={\lambda }_{p}\frac{\left[{V}_{f}^\frac{2}{3}+\frac{{\lambda }_{p}}{{\lambda }_{f}}\left(1-{V}_{f}^\frac{2}{3}\right)\right]}{\left[{V}_{f}^\frac{2}{3}-{V}_{f}+\frac{{\lambda }_{p}}{{\lambda }_{f}}\left(1-{V}_{f}^\frac{2}{3}+{V}_{f}\right)\right]}$$

where λ is the thermal conductivity of the composite material, λ_p_ is the thermal conductivity of the uniformly distributed filling phase, λ_f_ is the thermal conductivity of pores (air), and V_f_ is the volume fraction of spherical filling phase.

Taking 5% of the mass fraction of hollow glass beads as an example, the density is 0.35 g /cm^3^; the cement powder dosage is 600 g, the density is 3.5 g/cm^3^; the water addition amount is 347 g, the density is 1 g/cm^3^. The equivalent volume fraction is 13.75%. These parameters are substituted into the model for calculation, while the results are shown in Table 5. It can be seen that the prediction performance of Maxwell model for thermal conductivity is better than Russell model. Considering the simplification of the model using the aforementioned formula, it can be considered that the actual situation of composite materials is basically consistent with Maxwell model.

**Table 5:** Evaluation of Maxwell heat-conduction model for composites.

	Glass bead dosage	5%	10%	15%	20%
	Equivalent volume V_f_/%	13.75	23.65	31.02	36.73
Maxwell heat conduction model	Theoretical value of thermal conductivity/W·(m·K)^−1^	0.6285	0.5543	0.5024	0.4639
	Measured value of thermal conductivity/W·(m·K)^−1^	0.6704	0.6084	0.5591	0.479
	Prediction error	6%	9%	10%	3%
Russell heat conduction model	Theoretical value of thermal conductivity/W·(m·K)^−1^	0.6280	0.4779	0.3433	0.3338
	Measured value of thermal conductivity/W·(m·K)^−1^	0.6704	0.6084	0.5591	0.479
	Prediction error	6%	21%	39%	30%

#### Analysis and evaluation of foam cement heat conduction model

Maxwell model shows a good performance in predicting the thermal conductivity of composite with spherical filler. The prediction error is about 10% when the filler is hollow glass beads. The foam of the foamed cement prepared with chemical nitrogen-filled method has a uniform size distribution and good sphericity, thus it is suitable for Maxwell model. According to formula (2), the key to accurately predict the thermal conductivity is to determine the volume fraction of spherical filling phase. For glass beads, the volume fraction can be calculated by dosage and density. The gas filling in foamed cement slurry is nitrogen, and the compressibility is affected by temperature and pressure. Therefore, different methods should be taken into account for calculation.

For hardened foamed cement, the filling phase is nitrogen and the filling matrix is hardened cement, the thermal conductivity can be measured using the instrument. As such, the filling volume V_f_ of nitrogen can be calculated. The actual gas production of NGR can be measured experimentally, thus, the model can be verified. Combined the aforementioned assumptions with experimental results, the theoretical mass reduction of the system after foaming can be calculated as *ΔM* = V_total nitrogen_· ρ _nitrogen_ (1 − V_f_); By weighing the system mass before and after the experiment, whether the reduced mass of the system is in the theoretical calculation range can be verified. The corresponding summary is shown in Table 6.

**Table 6:** Validation data calculated by model inversion.

Actual thermal conductivity of hardened cement /W·(m·K)^−1^	Thermal conductivity of filling phase (in nitrogen) /W·(m·K)^−1^	Thermal conductivity of filling matrix /W·(m·K)^−1^	V_f_ theoretical value/%	Escape mass Δm/g
0.3933	0.023	0.7877	37–47	0.999–0.745

Combined with the experimental data of hollow glass beads, the error of 10% is taken according to the results of model calculation.

According to the aforementioned analysis, repeat the slurry preparation experiment and weigh the reaction vessel. After full reaction, the first weighing will be carried out (0.36 g) when the container returns to normal pressure, and the second weighing will be conducted after three minutes, which is 0.91 g. Based on that, V_f_ of nitrogen can be calculated as 43%, and the final predicted thermal conductivity of cement can also be calculated as 0.3845 W·(m·K)^−1^. The calculation shows that the escape mass is within the range of theoretical calculation. Considering the simplification and measurement error of the corresponding model, it can be considered that this model has good application potential in the foamed insulation cement slurry system prepared with chemical nitrogen-filled method.

#### Evaluation of thermal conductivity model for multicomponent composites

Although this model is suitable for the aforementioned cement slurry. However, due to the existence of glass bead filling into the foam, multicomponent composites can be regarded as an equivalent filling matrix as a whole when the amount of a certain material increases to a certain value. On this basis, the relationship between the addition amount of this material and the thermal conductivity can be studied. Based on the analysis mentioned above, and combined with the relevant data and actual condition of this experiment, the thermal conductivity of hardened cement with micro bead addition of 10% and 12.5% can be regarded as a recalculation of 7.5% addition, as shown in Table 7. It can be seen that the equivalent method can be used to simplify the calculation of thermal conductivity of multicomponent composites. Considering that the water cement ratio in the two groups of experiments is changed and that the error of the model itself exists, it can be considered that the actual situation of multicomponent composites is basically consistent with the theoretical model.

**Table 7:** Evaluation of thermal conductivity model for multicomponent composites.

Equivalent matrix thermal conductivity/W·(m·K)^−1^	Equivalent filling volume V_f_ (%)	Theoretical thermal conductivity/W·(m·K)^−1^	Measured thermal conductivity /W·(m·K)^−1^	Prediction error (%)
0.3973	10.1	0.3626	0.3561	1.8
0.3561	20.3	0.3056	0.2998	2

### New foam-bead thermal insulation cement slurry and its application

#### Cement slurry used in field

The buried depth of Ordovician carbonate reservoir in Tahe Area is larger than 5400 m, while the crude oil in this area is super heavy crude oil with high viscosity, high sulfur and high wax content. The existing wellbore has poor thermal insulation capacity and the heavy oil flowing in wellbore shows high heat loss, resulting in increased crude oil viscosity and its reduced flow capacity. This causes low drainage and production rate of heavy oil and exerts higher requirements for wellhead heating and dilution for enhanced production of heavy oil. In order to improve the efficiency of heavy oil development, it is urgent to strengthen the wellbore thermal insulation performance. By introducing foam and glass bead to cement slurry, the thermal conductivity of hardened cement is reduced, and a thermal insulation layer is formed on the periphery of the wellbore to reduce the heat loss rate during heavy oil wellbore movement. As such, heavy-oil temperature at the wellhead is improved, and the heavy-oil recovery is enhanced. From January to November 2021, 5 wells were applied on site.

Using the Class-G cement and with a water cement ratio of 0.44–0.55, the chemical nitrogen-filled is realized through the NGR. The foam stabilizer SC-1 is used to stabilize the foam, and the hollow glass bead is used to reduce the thermal conductivity of the hardened cement. The rheological properties, thickening time and water loss of cement slurry is further improved by adding proper amount of sulfonated aldehydes and ketones dispersant QS-20S, polymer retarder HX-36L, polyvinyl alcohol type water-loss reducer QS-12S, and nano-stabilizer NS-2. Chemical formulation of the new foam-micro bead thermal insulation cement slurry: Class-G cement + 2% Foaming agent NGR + 1.2% Foam stabilizer SC-1 + 12.5% glass beads + 0.3% dispersant QS-20S + 1.0% water loss additive QS-12S + 2.0% retarder HX-36L% + 2.0% nano stabilizer NS-2 + 44% water (Class-G cement quality as the benchmark).

The results of cement performance test are shown in Table 8. It can be seen that the fluidity of the novel insulation slurry system is between 20 and 23 cm and it exhibits strong flow capacity. The separated water ratio is zero, and the stability of the system is good; The water loss is within 50 mL/30 min, corresponding to a very good water-loss control ability; The thickening time is greater than 200 min, which is reasonable and can ensure the fluent cement slurry injection; The compressive strength cured for 72 h at 40 °C is greater than 15 MPa.

**Table 8:** Performance test of new foam-bead thermal insulation cement slurry.

Densityg/cm^3^	Water separation rate/%	API water loss /mL	Fluidity/cm	Thickening time /min (40 °C × 10 MPa)	Compressive strength/MPa (40 °C × atm)		Thermal conductivity (40 °C × atm) /W·(m·K)^−1^
					48 h	72 h	
1.25	0	32	22	205	15.80	18.46	0.3978

#### Field tests

5 wells were tried on site from January to November 2021. All the heavy oil wells in this block are tertiary structural wells, and the insulation cement slurry system was used in the cementing for the second opening. The well structure and structure parameters are shown below in Table 9.

**Table 9:** Well structure parameters of well LXP15.

Process	Bits		Casing		Note
	Size/mm	Drilling depth/m	Size/mm	Depth/m	
Fist opening	346.1	1200	273.1	1199.16	Conventional cementing
Second opening	241.3	5700–6200	193.7	(5700–6200)-2	Location of double-stage collar: 4000 m

In the field test, chemical-induced nitrogen foam cement slurry is uniformly produced by mixing cement base slurry and foam base fluid in the mixer. Double pump is used to inject cement slurry, and single pump is adopted to inject foam base fluid. The injection displacement of cement slurry is 1.5–2 m^3^/min, while the cement slurry and foam base fluid are pumped in a volume ratio of 10:1. Thus, the foam base fluid injection volume is around 0.1–0.2 m^3^/min, and displacement volume is about 1.8–2.4 m^3^/min. In the field operation, all the composite insulation cement slurry is successfully returned to the ground, and the cementing-quality good rate of 5 wells is 100%. In order to evaluate the thermal insulation effect of cementing wells, the wellhead temperature of 21 adjacent wells are selected for comparison, and the average wellhead temperature is 35.23 °C. The average wellhead temperature of the wells using thermal insulation cement is 47 °C, which is 11.77 °C higher than that of adjacent wells. Thus, the thermal insulation cement developed in this study plays a good role in wellbore thermal insulation.

## Conclusions

In this paper, a multi-material and multi-structure insulating cement slurry is developed, and a thermal-conductivity prediction method is proposed correspondingly based on Maxwell model.

1. A chemical-induced nitrogen-filled foamed cement is developed. The effects of foaming agents, hollow glass beads and their combinations on the compressive strength and thermal conductivity of hardened cement are analyzed. The optimal combination of chemical additives is obtained to balance the cement-slurry rheology, compressive strength and the thermal conductivity of hardened cement.
2. The reliability of the Maxwell model to predict thermal conductivity of cement filled with foam and glass beads is verified. The experimental results show that the prediction error is only 2%.
3. A new foam and glass bead-filled thermal insulation cement slurry system is developed. Its properties are list below: there is no free liquid, water loss is 32 mL/30 min, the 48 h strength is greater than 15 MPa, the 72 h compressive strength is more than 18 MPa, the thermal conductivity is 0.3719 W·(m·k)^−1^; Its performance could meet the requirements of in-situ geothermal well cementing, and could significantly reduce the heat loss in geothermal production process.
4. The newly developed cement slurry is applied to the cementing test for heavy oil insulation in Xinjiang ultra-deep well of China. For the 5 trial wells, the cementing quality good rate is 100%. The wellhead temperature of the wells using the new cement is 11.77 °C higher compared with the 21 adjacent wells using fly-ash low density cementing. Field application shows that the thermal insulation cement slurry system can provide high thermal insulation effect on the premise of ensuring cementing quality, and provide a new technology for efficient exploitation of ultra-deep heavy oil and efficient utilization of geothermal resources in the future.

## Data Availability

All data generated or analysed during this study are included in this published article. And the datasets used and/or analysed during the current study available from the corresponding author on reasonable request.
